# An analysis of the selection criteria for postgraduate physician assistant residency and fellowship programs in the United States

**DOI:** 10.1186/s12909-021-03059-y

**Published:** 2021-12-16

**Authors:** Vasco Deon Kidd, Sarah Vanderlinden, Jennifer M. Spisak

**Affiliations:** 1 School of Medicine, Department of Orthopaedic Surgery, University of California Irvine (UCI Health), 101 The City Dr S, Orange, CA 92868 USA; 2 Department of Surgery, Trauma and Critical Care, Medical College of Wisconsin, Milwaukee, WI USA; 3 Ronald O. Perelman Department of Emergency Medicine, NYU Langone Health, 545 First Avenue, Greenberg Hall Suite 6B, New York, NY 10016 USA

**Keywords:** Physician assistant, Physician associate, Nurse practitioner, Residency, Fellowship, Advanced practice provider, Postgraduate training, Admission criteria

## Abstract

**Background:**

This study aims to investigate the admission criteria used by physician assistant postgraduate education programs in selecting licensed PA applicants for postgraduate training in the United States. To our knowledge, there have been no previously published reports on selection criteria and/or other factors influencing postgraduate PA admission decisions.

**Method:**

A non-experimental, descriptive research study was designed to obtain information from members of the Association of Postgraduate Physician Assistant Programs (APPAP).

**Results:**

Twenty-three out of 73 postgraduate programs (31.5%) responded to the survey. The study reported that applicant PAs and nurse practitioners (NPs) are largely selected on the basis of several factors. The most heavily weighted factor is the interview itself; other selection criteria perceived to be *extremely/very important* included board certification/eligibility, letters of recommendation, advanced degree, and personal essay. Survey data suggest that publications, undergraduate transcripts, and class rankings are not considered to be of high importance in applicant selection.

The number of PA applicants applying to each postgraduate training program averages around 26 and total number of enrollees is about 3.6 per program. Additionally, some programs reported furloughing of trainees (temporary suspension of didactic and clinical training) during the pandemic, whereas the vast majority of postgraduate PA programs remained operational and some even experienced an increase in application volume. The total cost of training a PA resident or fellow in postgraduate programs is currently $93,000 whereas the average cost of training a categorical physician resident is estimated at $150,000 per year when considering both salary and benefits.

**Conclusions:**

This novel study examined criteria and other factors used by postgraduate PA programs in selecting candidates for admission. Results can be used by postgraduate programs to improve or modify current selection criteria to enhance the quality of trainee selection. Further research is needed to examine correlations between applicant attributes, selection criteria, and trainee success in completing postgraduate training.

**Supplementary Information:**

The online version contains supplementary material available at 10.1186/s12909-021-03059-y.

## Background

The development of postgraduate programs for physician assistants (PAs) began over four decades ago [1, 2]. Currently, there are approximately 73 postgraduate programs affiliated with the Association of Postgraduate Physician Assistant Programs (APPAP) spread across a broad range of medical and surgical disciplines [1, 2]. All member programs of APPAP are formal postgraduate PA programs that offer structured curricula, including didactic and clinical components, to educate licensed and certified PAs for a defined period of time (usually 12 months) in a medical specialty (APPAP.org). Given that PAs are trained as generalists in the medical model, postgraduate programs provide PAs with specialty educational experience. However, factors influencing specialty or subspecialty choice of postgraduate training among PAs is not well elucidated in the literature. Unlike resident categorical training, there is no board certification available for PAs who complete a postgraduate training program. Moreover, 98% offer a certificate of completion upon graduation. While formal accreditation by the Accreditation Review Commission on Education for the Physician Assistant (ARC-PA) is available as of 2020, it remains optional for postgraduate training programs.

When it comes to PA postgraduate selection admission criteria, there is no published data on which factors used by postgraduate programs most affect the admission decision. Therefore, the purpose of this study is to determine the selective admission criteria that are important to postgraduate programs in selecting applicants for medical or surgical specialty training.

## Method

A non-experimental, descriptive research study was designed to obtain information from members of the Association of Postgraduate Physician Assistant Programs (APPAP). The list of programs was drawn from an updated APPAP membership page (APPAP.org). After review of the literature and based on the substantive experience of the authors in postgraduate PA education, an online survey was developed. The survey was comprised of 22 items and after a successful pilot, the survey was finalized. The three respondents who took part in the pilot test did not participate in the overall survey to limit bias. Individual postgraduate programs were sent an email invitation and a link to a voluntary, anonymous online survey. The email introduction to the survey contained all the necessary elements of written consent, and submission of the survey indicated the respondents’ consent to participate. The study period was from 8/17/2021 through 9/17/2021. Five reminders were sent to postgraduate programs during the 30-day study period. The participants completed the survey through the online tool SurveyMonkey. Confidentiality was maintained throughout the study and no identifying information was recorded. Survey responses were aggregated, and descriptive statistical analyses were utilized through a statistical package embedded within the survey software. The survey was reviewed with the University of California Irvine (UCI) IRB team and the study is exempt under the UCI Exempt Self-Determination Tool. As part of using the Exempt Self-Determination Tool, Lead Researchers and Faculty Sponsors (as applicable) provided their assurance that they followed relevant Human Research Protection Program (HRPP) policies and procedures, among other criteria.

## Results

Seventy-three postgraduate programs were invited to participate, and 23 programs completed the entire survey. The overall response rate was 31.5%. Response rates varied by specialty: emergency medicine (34%), general surgery (13%), critical care medicine (8%), orthopaedic surgery (8%), neonatology (8%), cardiothoracic surgery (8%), psychiatry (4%), obstetrics and gynecology (4%), internal medicine (4%), and trauma and surgical critical care (4%). Of those that completed the survey, 87% were program directors, 9% associate program directors, and 4% were NPs affiliated with the program. Additionally, (34%) of respondents enroll both PAs and NPs, whereas (65%) only enroll PAs.

Selection criteria perceived as *extremely important* by postgraduate programs included personal interview (96%), board certification/eligibility (83%), letters of recommendation (61%), graduate degree (52%), and personal essay (44%). Perceived as *very important*: clinical rotation grades (44%), awards/achievements (44%), transcripts from PA or NP entry level program (44%), and overall GPA from PA or NP school (39%). Perceived as *importan*t: community service and class ranking (48%). Perceived as *somewhat/not important*: publications (78%), membership/position in a local PA or NP association (52%), and transcripts from undergraduate study (52%) (Table 1). The majority of respondents (86%) reported that their selection criteria can be found on their website, while 27% of programs include selection criteria on the website, job description, and brochure.

**Table 1:** Importance of application criterion in selecting postgraduate PA residency and fellowship program candidates

	Extremely Important	Very Important	Important	Somewhat Important	Not Important
Personal Interview	96%	4%	0%	0%	0%
Board Certification Eligibility	83%	4%	9%	4%	0%
Letters of Recommendation	61%	30%	9%	0%	0%
Graduate Degree	52%	22%	0%	9%	17%
Personal Essay	44%	52%	4%	0%	0%
Clinical Rotation Grades	13%	44%	35%	4%	4%
Achievements/Awards	13%	44%	26%	17%	0%
PA or NP Program Transcripts	9%	44%	44%	3%	0%
GPA in PA or NP School	9%	39%	39%	13%	0%
Community Service	4%	22%	48%	17%	9%
Class Ranking	0%	9%	48%	17%	26%
Undergraduate Transcripts	0%	22%	26%	22%	30%
Membership in PA or NP Association	0%	13%	35%	17%	35%
Publications	0%	0%	22%	30%	48%

Total program response was *N* = 23

### Admission cycle and screening process

Programs were asked about their application cycle and reported a fixed deadline admission process (82%) or rolling admission process (17%); however, application and selection deadlines vary among postgraduate programs as there is no centralized application processing service available to PA and NP postgraduate applicants. A majority of respondents do not charge an application fee (60%). Respondents reported that participants involved in the screening process include the program director (91%), associate program director (47%), medical director (26%), and human resources business partner (13%).

### Candidate interviewing

The program personnel involved in interviewing applicants comprises a diverse interview panel and includes the program director (100%), medical director (69%), associate program director (52%), staff PAs/NPs (52%), physicians (34%), and to a lesser extent, resident physicians and human resource recruiters (17%). Moreover, postgraduate program interviews come in many different forms such as structured interview (60%), panel interview (39%), and multiple mini interview (34%). During the interview, most programs included a facility tour (95%) and introduction to program staff (73%), while other programs reported that candidates participated in a group activity (39%). Nearly (20%) evaluated candidates with a pre-admission assessment.

### Candidate selection

**Fig. 1:**
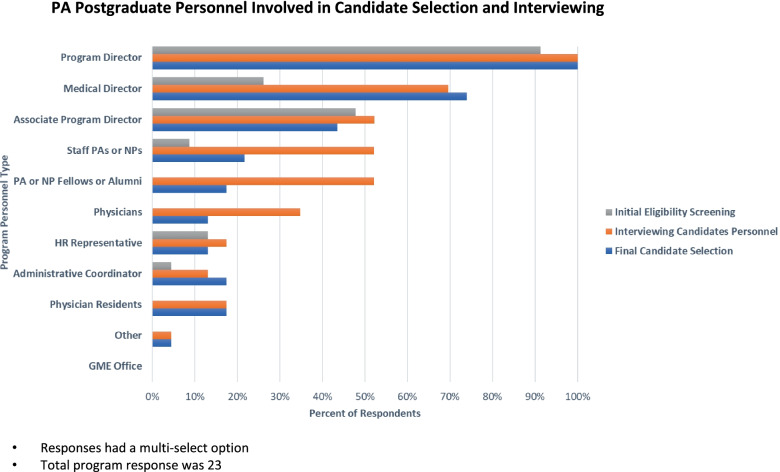
PA Postgraduate Personnel Involved in Candidate Selection and Interviewing. Responses had a multi-select option. Total program response was 23

The data from our study suggest that postgraduate programs include multiple stakeholders in selecting candidates for admission. Respondents reported program personnel responsible for making the final admission decision included the program director (100%), medical director (73%), associate program director (43%), and staff PAs/NPs (21%) (Fig. 1).

### COVID-19 pandemic impact

The majority (96%) of postgraduate programs were operational during the evolving COVID-19 pandemic, however, 22% of programs reported that their trainees experienced furlough; the length of the furlough was not assessed in our study. Approximately 4% of programs experienced closure. Additionally, 70% of programs reported an increase in the number of applicants without a corresponding increase in enrollees. Lastly, 26% of postgraduate programs experienced a decrease in program funding.

## Discussion

The goal of this research study was to investigate admission criteria used by PA postgraduate programs in selecting licensed PA applicants for postgraduate training in the United States. There are no published data detailing factors used by postgraduate programs to drive admission decisions.

This study revealed the influence and weight of particular selection criteria as well as the stakeholders evaluating that criteria. Criteria driving the selection process include personal interview, board certification/eligibility, letters of recommendation, achievement of a graduate degree, and personal essay. Least important to key stakeholders, namely program directors, but also medical directors, associate program directors, and staff PAs/NPs was participation in research/publication, membership in local PA/NP associations, and undergraduate transcripts.

Aside from board certification/eligibility and achievement of a graduate degree, the factors deemed most important of the selection criteria for admission to a postgraduate PA program are subjective in nature. The weight placed on the personal interview, personal essay, and letters of recommendation supersedes that of graduate GPA, class rank, and clinical rotation grades, suggesting that applicant characteristics of personability, interpersonal skills, communication skills, emotional intelligence, and adaptability are as important to demonstrate as clinical acumen via board certification. This is not too dissimilar from how physician residency programs evaluate certain characteristics in the selection of candidates for postgraduate training [3]. Importantly, how these non-cognitive attributes were conveyed over a virtual platform must be considered, as interviews were primarily conducted virtually during the COVID-19 pandemic.

According to published research, 36% of physician residency programs cited “demonstrated involvement and interest in research” as a factor when granting applicants an interview [4]; however, our study demonstrated a lower importance on research/publications of PA postgraduate education applicants. This may reflect the historical focus of PAs on clinical care (i.e., application of knowledge vs. discovery of knowledge) in the curriculum of their training and their subsequent professional practice, but also serves to highlight the scant contribution of PAs to the field of medical research that has been previously observed [5]. Anecdotally, many postgraduate applicants are new graduates, with little or no research experience, which may reflect a knowledge gap and lack of research mentorship.

Barriers to research contribution have previously been considered and include lack of experience with research, lack of time, lack of interest, lack of mentors and lack of funding [5]; data from this study suggest that these barriers need to be addressed at both the graduate and postgraduate levels especially if the goal is to bring greater attention and awareness to increasing the number and caliber of PA research contributors [6, 7]. A recent published study suggested that improving research methodology training and having protected time for research are strategies that may lead to increased scholarly productivity in postgraduate residencies [8].

Also, of little importance to postgraduate admissions was membership in a PA/NP association. Encouraging participation in local and national organizations should be a priority at both the graduate and postgraduate level to increase advocacy of the PA profession from career inception. Preparing future advocates to advance the profession will serve to fortify the PA presence as key stakeholders in health care advancement at local, national, and international levels.

Additionally, this study identified program personnel involved in the admission process, from screening applications to conducting interviews and making applicant selections, indicating that the program director primarily, along with associate program directors and medical directors had the greatest influence on the interview process and selection of candidates for admission. It should be noted that the graduate medical education (GME) office was not found to be involved in the screening, interviewing, or selection of candidates for postgraduate PA training. This distinction suggests that, while housed in the same institution, PA postgraduate education processes and GME education processes are two separate entities with different funding, goals, and resources.

Throughout the COVID-19 pandemic, the vast majority of postgraduate PA programs remained operational, while many saw an increase in application volume. The uptick in applications may have been attributed to a perceived skills gap among recent PA and NP entry level graduates who experienced limited face-to-face clinical education (clinical clerkship/practicums) during their training or encountered difficulty in securing employment subsequent to graduation as a consequence of the COVID-19 pandemic. Additionally, a quarter of postgraduate programs experienced a decrease in program funding. This may be largely due to financial losses exacerbated by COVID-19 in which US hospitals now face a liquidity crisis; some institutions may have elected to cut back on funding for training programs that they deem a drain on already limited resources [9].

Lastly, the estimated cost (salary/benefits) of training PA and NP postgraduate fellows is around $93,000. This is the first study to provide a reasonable estimate of typical costs associated with PA and NP postgraduate training. Awareness of the costs of training will help shed light on the financial contribution of the sponsoring institution to develop or sustain new and existing postgraduate PA programs.

### Limitations

Our study is not without limitations. First, this is survey data and only members of APPAP were surveyed. Post-graduate programs are heterogeneous in nature so this sample may not represent the wide diversity of programs. Second, we did not evaluate the impact of racial, geographical, or gender differences among applicants during program selection. Third, this study includes a low response rate of (31.5%), which may have contributed to response bias. Fourth, overrepresentation of emergency medicine programs may also have led to some bias and the following specialties in which PA postgraduate training is available but underrepresented in this survey includes transplant surgery, cardiology, geriatrics, hospice and palliative care, structural heart, hepatobiliary surgery, plastic surgery, vascular surgery, urology, urgent care, pediatric medical specialties, pulmonology, primary care including rural medicine. Lastly, when programs were asked to report on the total cost (salary/benefits) of training a PA resident or fellow, five programs reported only salary costs. Therefore, we multiplied the salary cost by the 32% benefit rate (Bureau of Labor Statistics) as a percentage of salary (2018) to determine total postgraduate cost of training of a PA and NP in those 5 programs. [10]. Despite some limitations, our study highlights differences in selection criteria by postgraduate PA programs in the initial screening of applicants and subsequent selection of candidates for specialty training.

## Conclusion

This study presents the admissions practices of PA postgraduate education programs in the US. The data suggest that these postgraduate programs use a multifactorial application process in accepting candidates for specialty training. The data from this study can help guide and improve selection practices across postgraduate education programs as well as inform future applicant and policy decisions.

### Areas of future research

Future research is needed to examine correlations between applicant attributes, selection criteria, and trainee success in completing postgraduate training. Another area of exploration is whether postgraduate education programs closely align with employment and/or academic institutional priorities. Future investigation of postgraduate training should also include evaluation of other fixed costs not highlighted in this study, such as program personnel and technology costs, faculty development expenses, and accreditation fees. Lastly, research is needed to explore the main factors (work/life balance, lifestyle, clinical interest, personality fit, income expectations, etc) influencing the choice of graduate PAs in pursuing a specialty postgraduate training program.

## Supplementary Information

**Additional file 1.**

**Additional file 2.**

## Data Availability

Available from the corresponding author and APPAP Survey committee on reasonable request.
