# Correction to: An analysis of the selection criteria for postgraduate physician assistant residency and fellowship programs in the United States

**DOI:** 10.1186/s12909-022-03184-2

**Published:** 2022-02-18

**Authors:** Vasco Deon Kidd, Sarah Vanderlinden, Jennifer M. Spisak

**Affiliations:** 1 School of Medicine, Department of Orthopaedic Surgery, University of California Irvine (UCI Health), 101 The City Dr S, Orange, CA 92868 USA; 2 Department of Surgery, Trauma and Critical Care, Medical College of Wisconsin, Milwaukee, WI USA; 3 Ronald O. Perelman Department of Emergency Medicine, NYU Langone Health, 545 First Avenue, Greenberg Hall Suite 6B, New York, NY 10016 USA

**Correction to: BMC Med Educ 21, 621 (2021)**

**https://doi.org/10.1186/s12909-021-03059-y**

Following publication of the original article [1], the author informed us that the response rate was incorrectly reported. The updated rate is given below and the changes have been highlighted in **bold typeface** as follows:

**Abstract**

**Results:** Twenty-three out of 73 postgraduate programs **(31.5%)** responded to the survey. The study reported that applicant PAs and **nurse practitioners (NPs)** are largely selected on the basis of several factors.

**Results**

Seventy-three postgraduate programs were invited to participate, and 23 programs completed the entire survey. The overall response rate was **31.5%.**

**Limitations**

Third, this study includes a low response rate of **(31.5%)**, which may have contributed to response bias.

The original article has been corrected.
