# Supplementary material for: An analysis of the selection criteria for postgraduate physician assistant residency and fellowship programs in the United States
Source: BMC Med Educ. 2021 Dec 16;21:621. doi: 10.1186/s12909-021-03059-y (PMC8675298; doi:10.1186/s12909-021-03059-y)
Supplement: Supplementary file 1 — Additional file 1. [file 12909_2021_3059_MOESM1_ESM.pdf]

APPAP
The Association of Postgraduate PA Programs

APPAP 2021 Survey

What are common criteria used by postgraduate PA programs in selecting candidates for admission?

An analysis of the selection criteria for postgraduate PA Residency/Fellowship Programs in the US.

1. What is your postgraduate program's specialty?

2. What is your current title?

- ◯ Program Director
- ◯ Associate Program Director
- ◯ Medical Director
- ◯ Other (open textbox)

3. Are you a:

- ◯ PA
- ◯ NP
- ◯ MD / DP

4. During the COVID-19 pandemic, did your program experience:

	Decrease	No Change	Increase
Change in number of program enrollees?	◯	◯	◯
Change in applications?	◯	◯	◯
Change in program funding?	◯	◯	◯
Change in number of applications?	◯	◯	◯

5. During the COVID-19 pandemic, did your program experience:

	Yes	No
Furlough of enrolled APP trainees?	◯	◯
Program Closure?	◯	◯

6. In addition to PAs, does your program accept NPs?

- ◯ Yes
- ◯ No

7. How would you classify your admission process?

- ◯ Rolling admission
- ◯ Fixed-deadline admission

8. Where is the program's selection criteria located?

- ☐ Website
- ☐ Job Description
- ☐ Brochure
- ☐ All of the Above
- ☐ Other (please specify)

9. Do you charge an application fee?

- ◯ Yes
- ◯ No

10. Who is responsible for initially screening applications for eligibility? (Select all that apply

- ☐ Program Director
- ☐ Associate Program Director
- ☐ Medical Director
- ☐ Clinical or administrative coordinator
- ☐ Physicians
- ☐ Staff PAs/NPs
- ☐ HR Recruiter/Representative
- ☐ GME Office
- ☐ Other (please specify)

11. Which of the following program personnel is involved in interviewing applicants? (Select all that apply.)

- ☐ Program Director
- ☐ Associate Program Director
- ☐ Medical Director
- ☐ Clinical or Administrative Coordinator
- ☐ Physicians
- ☐ Physician Resident
- ☐ Staff PAs/NPs
- ☐ PA or NP Fellows/Residents or Alumni
- ☐ GME Office
- ☐ HR Recruiter/Representative
- ☐ Other (please specify)

12. The applicant interview process is a: (Select all that apply)

- ☐ Structured interview process, which relies on a set of standardized and premeditated questions.
- ☐ Unstructured interview process, which does NOT rely on a set of standardized or Premediated questions.
- ☐ Multiple Mini Interviews (MMI), which assesses non-cognitive qualities including cultural sensitivity, maturity, teamwork, empathy, reliability and communication skills.
- ☐ Panel interviews
- ☐ Other (please specify)

13. Does your interview process include any of the following? (Select all that apply)

- ☐ In-person or virtual tour of the facilities
- ☐ Group activity
- ☐ Introduction to program staff
- ☐ Pre-admission assessment
- ☐ Other (please specify)

14. Do you offer candidates virtual interviews, if needed?

- ◯ Yes
- ◯ No

15. Which of the following program personnel is responsible for making the final decision of admission to the program? (Select all that apply)

- ☐ Program Director
- ☐ Associate Program Director
- ☐ Medical Director
- ☐ Clinical or Administrative Coordinator
- ☐ Physicians
- ☐ Staff PAs/NPs
- ☐ GME Office
- ☐ PA and/or NP Fellows/Residents
- ☐ Other (please specify)

16. Please rank the level of importance of each of the following selection criterion?

	Extremely Important	Very Important	Important	Somewhat Important	No Important
Board Certification/Eligibility	◯	◯	◯	◯	◯
Letters of Recommendation	◯	◯	◯	◯	◯
Personal Interview	◯	◯	◯	◯	◯
Publications	◯	◯	◯	◯	◯
An Advanced Degree (ie, Master's Degree)	◯	◯	◯	◯	◯
Personal Essay	◯	◯	◯	◯	◯
Membership/Position in a local PA or NP Association	◯	◯	◯	◯	◯
Clinical Rotation Grades	◯	◯	◯	◯	◯
Transcripts from PA or NP program	◯	◯	◯	◯	◯
Transcripts from Undergraduate Study	◯	◯	◯	◯	◯
Community Service	◯	◯	◯	◯	◯
Overall GPA from PA or NP school	◯	◯	◯	◯	◯
Class Ranking	◯	◯	◯	◯	◯
Achievements/Awards	◯	◯	◯	◯	◯

17. Does your postgraduate program notify admitted fellows/residents that they are eligible for loan deferment.

- ◯ Yes
- ◯ No
- ◯ Not sure

18. How many complete PA applications do you receive annually (total for all recruitment cohorts)?

19. How many complete NP applications do you receive annually (total for all recruitment cohorts)?

20. How many total enrollees do you accept annually?

21. What is the total cost (salary/benefits) budgeted to train a single PA and/or NP in your program?

22. Additional comments about your recruitment process:

Done

Powered by
SurveyMonkey
See how easy it is to create a survey.

Privacy & Cookie Policy
