# Supplementary material for: An analysis of the selection criteria for postgraduate physician assistant residency and fellowship programs in the United States
Source: BMC Med Educ. 2021 Dec 16;21:621. doi: 10.1186/s12909-021-03059-y (PMC8675298; doi:10.1186/s12909-021-03059-y)
Supplement: Supplementary file 2 — Additional file 2. [file 12909_2021_3059_MOESM2_ESM.pdf]

UCI University of California, Irvine

Office of Research
160 Aldrich Hall
Irvine, CA 92697-3175
Front Office (949) 824-7295 | Fax (949) 824-3400 |research.uci.edu

THE EXEMPT SELF DETERMINATION PROCESS

March 2021

To Whom it May Concern:

The Exempt Self-Determination Tool may be used for self-determining certain types of exempt research at UCI, including exempt research conducted through the Undergraduate Research Opportunities Program (UROP). Exceptions do apply. Please refer to UCI HRPP Policy # 12 for the current exceptions.

As part the Exempt Self-Determination Tool process, UCI IRB review is not required and will not be provided. For studies that are submitted to the IRB where the Exempt Self-Determination Tool may be used instead, the study will be returned to the researcher to self-exempt. For exempt studies that require UCI IRB review, Lead Researchers must submit an IRB application and supporting documents to the UCI IRB for review.

UROP students using the Exempt Self-Determination Tool to conduct exempt research should contact UROP for questions related to the use of the tool.

As part of using the Exempt Self-Determination Tool, Lead Researchers and Faculty Sponsors (as applicable) provide their assurance that they will follow relevant Human Research Protection Program (HRPP) policies and procedures, among other criteria. For a copy of the assurance, please review the following page.

If there are any questions regarding UCI HRPP Policy # 12, please contact HRPP Staff.

-The UCI HRPP

Office of Research
160 Aldrich Hall
Irvine, CA 92697-3175
Front Office (949) 824-7295 | Fax (949) 824-3400 |research.uci.edu

AS PART OF THE EXEMPT SELF DETERMINATION PROCESS AT UCI, THE LEAD RESEARCHER AND FACULTY SPONSOR (AS APPLICABLE) ASSURES THE FOLLOWING:

1. The information provided in this application is accurate to the best of my knowledge.

2. All named individuals on this project have read the procedures outlined in the protocol, are aware of and have reviewed relevant HRPP Policies and Procedures and understand their role on the study.

3. All named individuals on this project have completed the required electronic educational research tutorials and have been made aware of the "Common Rule" (45 CFR Part 46) and acknowledge the importance of the Belmont Principles - Respect for Persons, Beneficence and Justice in conducting research involving human participants. Also UCI has signed the Federalwide Assurance (FWA) that is available for review on the Human Research Protections (HRP) website.

4. Minor changes to the research that do not increase risk to participants, or significantly alter the study aims or procedures, such as the addition or removal of students researchers, do not require additional self-confirmation of exemption or approval from the IRB. Major changes that increase risk or constitute substantive revisions to the research including procedural changes will require a new self-confirmation of exemption or approval from the IRB.

5. When conducting research off-site or collaborating with an investigator at another institution (e.g., another UC, CHOC, CSUF, or a local school district), Lead Researchers must comply with the requirements and policies of the site, including securing Confirmation of Exempt Status from the IRB.

6. The Exempt Self-Determination, consent documents including recruitment materials and data collection materials will be maintained by the Lead Researcher or Faculty Sponsor for 10 years beyond the completion of the research.

7. This research study is subject to routine monitoring by the Human Research Protections (HRP) unit of the Office of Research. Through the Education Quality and Improvement Program (EQUIP) program, HRP staff conduct periodic quality improvement monitoring and educational outreach.
